# Characterization and Interactome Study of White Spot Syndrome Virus Envelope Protein VP11

**DOI:** 10.1371/journal.pone.0085779

**Published:** 2014-01-21

**Authors:** Wang-Jing Liu, Hui-Jui Shiung, Chu-Fang Lo, Jiann-Horng Leu, Ying-Jang Lai, Tai-Lin Lee, Wei-Tung Huang, Guang-Hsiung Kou, Yun-Shiang Chang

**Affiliations:** 1 Department of Earth and Life Science, University of Taipei, Taipei, Taiwan; 2 Department of Molecular Biotechnology, Da-Yeh University, Changhua, Taiwan; 3 Institute of Bioinformatics and Biosignal Transduction, National Cheng Kung University, Tainan, Taiwan; 4 Institute of Marine Biology, National Taiwan Ocean University, Keelung, Taiwan; 5 Center of Excellence for the Oceans, National Taiwan Ocean University, Keelung, Taiwan; 6 Department of Food Science, National Quemoy University, Kinmen, Taiwan; 7 Institute of Zoology, National Taiwan University, Taipei, Taiwan; Uppsala University, Sweden

## Abstract

White spot syndrome virus (WSSV) is a large enveloped virus. The WSSV viral particle consists of three structural layers that surround its core DNA: an outer envelope, a tegument and a nucleocapsid. Here we characterize the WSSV structural protein VP11 (WSSV394, GenBank accession number AF440570), and use an interactome approach to analyze the possible associations between this protein and an array of other WSSV and host proteins. Temporal transcription analysis showed that *vp11* is an early gene. Western blot hybridization of the intact viral particles and fractionation of the viral components, and immunoelectron microscopy showed that VP11 is an envelope protein. Membrane topology software predicted VP11 to be a type of transmembrane protein with a highly hydrophobic transmembrane domain at its N-terminal. Based on an immunofluorescence assay performed on VP11-transfected Sf9 cells and a trypsin digestion analysis of the virion, we conclude that, contrary to topology software prediction, the C-terminal of this protein is in fact inside the virion. Yeast two-hybrid screening combined with co-immunoprecipitation assays found that VP11 directly interacted with at least 12 other WSSV structural proteins as well as itself. An oligomerization assay further showed that VP11 could form dimers. VP11 is also the first reported WSSV structural protein to interact with the major nucleocapsid protein VP664.

## Introduction

White spot syndrome virus (WSSV; genus *Whispovirus*, family *Nimaviridae* [1]) is an important crustacean viral pathogen that was been noted for its high virulence in most cultured shrimp and has caused severe mortality and huge economic losses to the word’s shrimp farming industry [2], [3]. WSSV is a large enveloped DNA virus approximately 275 by 120 nm in size and has a ∼300 kbp double-stranded DNA genome [4]. The WSSV virion has three structural layers surrounding its core DNA: an outer envelope, a tegument and a nucleocapsid [5], [6]. Proteomic methods have helped to show that the WSSV virion is composed of a total of more than 50 structural proteins [5]–[9].

In the virions of enveloped viruses, the structural proteins often form complexes, and some of WSSV’s structural proteins are also known to interact with other WSSV structural proteins. For example, VP28 interacts with VP24 and VP26 [8], [10], VP24 interacts with WSV010 [11], and VP90 interacts with VP26 and VP28 [12]. In our previous studies, we used pairwise protein-protein interaction analysis to investigate the interactions among several WSSV major structural proteins. And, with the help of additional external data, we determined the relationships between VP19, VP24, VP26, VP28, VP37, VP38A, VP51A, VP51C and WSV010, and then used this information to construct a model of the WSSV virion structural protein complex [13], [14]. However, a global analysis of WSSV structural proteins has not yet been published, and to date, our knowledge of the relationships among the WSSV structural proteins is still limited.

There is increasing evidence that WSSV structural proteins play very important roles in virus-host interactions. Several WSSV structural proteins involved in shrimp infection and host immune responses have been identified [10], [15]–[22], and some of these have been proposed as targets for developing new WSSV detection methods and anti-WSSV strategies [5], [10], [18]–[20], [23]–[26]. However, all of these studies have their limitations because they all studied each individual protein in isolation, whereas the protein’s interactome − i.e. its role and behavior in the context of its structural protein complex − was not considered.

In the present study, we characterize the WSSV structural protein VP11, which corresponds to ORF394 of the WSSV-TW strain (WSSV394, GenBank accession number AF440570). WSSV VP11 was named for its apparent size in a mass spectrometry study of WSSV virion proteins [7], but here we use Western blotting to show that its size is 38 kDa. VP11’s protein-protein interactions were determined by constructing a yeast two-hybrid prey library, consisting most of WSSV’s structural protein genes. This is the first study to use an interactome approach to analyze the possible associations between an individual WSSV structural protein and an array of other WSSV proteins.

## Materials and Methods

### Virus

The WSSV-TW strain was isolated from a batch of WSSV-infected *Penaeus monodon* collected in Taiwan in 1994 [4]. It was used here for the experimental viral inoculum and as the template for amplification of the coding regions of *vp11* as well as the other WSSV structural protein genes used in this study.

### Temporal Transcription Analysis of *vp11* by RT-PCR

Adult *P. monodon* (mean weight ∼20 g) were experimentally infected with WSSV by injection and subsequently collected at 0, (i.e., immediately before infection), 2, 4, 6, 12, 24, 36, 48 and 60 hours post infection (hpi) according to the procedure described by Tsai et al. [27]. Total RNAs were isolated from the gills of the sampled shrimp by using TRIzol reagent (Invitrogen Corp.) according to the manufacturer’s instructions. The isolated RNAs were treated with DNase I (Roche) at 37°C for 1 hour and then recovered by phenol-chloroform-isoamyl alcohol extraction and ethanol precipitation. The RNAs were reverse transcribed with SuperScript II reverse transcriptase (Invitrogen Corp.) and an oligo(dT) anchor primer (Roche). The first-strand cDNA products were then subjected to PCR with the *vp11* primers vp11-F1 and vp11-R1. For comparison, WSSV *ie1* (an immediate early gene), *dnapol* (a DNA polymerase gene) and *vp28* (an envelope protein gene) gene fragments were also amplified from the same templates using the primer pairs ie1-F/ie1-R, dnapol-F/dnapol-R and vp28-F/vp28-R, respectively [28]. An elongation factor 1-α gene (*EF1-α*) (GenBank accession number AF100987) primer pair, EF1-α-F1 and EF1-α-R1, was used as an internal control for RNA quality and amplification efficiency [29]. Sequences of the primers used here are listed in Table 1.

**Table 1:** Primer sequences used for RT-PCR.

Gene name	Primer sequences (5′-3′)
*vp11*	vp11-F1: GATGTCGGACAGGCAATTGC
	vp11-R1: CGGGCTTTTTCAATACGTCC
*ie1*	ie1-F: GACTCTACAAATCTCTTTGCCA
	ie1-R: CTACCTTTGCACCAATTGCTAG
*dnapol*	dnapol-F: TGGGAAGAAAGATGCGAGAG
	dnapol-R: CCCTCCGAACAACATCTCAG
*vp28*	vp28-F: CTGCTGTGATTGCTGTATTT
	vp28-R: CAGTGCCAGAGTAGGTGAC
*EF1-α*	EFI-α-F1: TCGCCGAACTGCTGACCAAGA
	EFI-α-R1: CCGGCTTCCAGTTCCTTACC

### Recombinant VP11 Protein Expression, Purification and Antibody Production

PCR fragments representing the amino acid 50–180 region of VP11 were amplified, digested with restriction enzymes, and cloned into pET-28b (+) (Novagen). The resulting plasmid, pET-28b/VP11_50−180_-His, was transformed into BL21 Codon Plus *Escherichia coli* cells (Stratagene) and used for protein production. The transformed cells were grown overnight at 37°C in a Luria-Bertani medium supplemented with kanamycin (50 µg/ml). The cells were then inoculated into a fresh medium at a ratio of 1∶50 and grown at 37°C for 1.5 to 2 hours. Expression was induced by the addition of 1 mM IPTG (isopropyl-β-D-thiogalactopyranoside), and incubation was continued for another 1.5 to 3 hours. The induced bacteria were spun down at 4°C, suspended in ice-cold 1× PBS containing 10% glycerol and a protease inhibitor cocktail tablet (Roche Applied Science), and sonicated for 3 minutes on ice. The insoluble debris was collected by centrifugation, suspended in 1× PBS containing 1.5% sodium lauryl sarcosine, and solubilized by shaking at room temperature for 1 hour. The suspension was then clarified by centrifugation and mixed with Ni-nitrilotriacetic acid-agarose beads (Qiagen) on a rotating wheel at 4°C for 16 hours. The beads were then washed several times with ice-cold wash buffer (1 M NaCl, 10 mM Tris-HCl, pH 7.5) to remove unbound material. The fusion proteins were eluted directly from the beads with SDS sample buffer and then subjected to SDS-PAGE analysis. For polyclonal antibody production from the recombinant VP11_50−180_, the protein bands containing the fusion proteins were sliced from the gel, minced, mixed with Freund’s adjuvant, and inoculated into rabbits. The specificity of the antibody was then tested by Western blotting performed on full-length recombinant VP11 that was expressed in Sf9 insect cells. To prepare the recombinant VP11, the full-length *vp11* coding region was inserted into a V5-tagged vector containing the heat inducible *Drosophila* heat shock protein 70 promoter (pDHsp/V5-His [29]) by PCR cloning, which used WSSV genomic DNA as the template. For DNA transfection, Sf9 cells were seeded onto a 4-well microplate (3×10^5^ cells/well) and grown in Sf-900 II serum-free medium (Invitrogen Corp.) overnight at 27°C. The *vp11*-containing plasmids, pDHsp/VP11-V5-His, were transfected into the Sf9 cells using Cellfectin reagent (Invitrogen Corp.). After transfection for 16–18 hours, the cells were heat shocked in a 42°C water bath for 30 minutes and then returned to 27°C. At 6 hours after heat induction, the cells were washed with 1× PBS and lysed in 100 µl of NP-40 lysis buffer (50 mM Tris-HCl, pH 8.0, 150 mM NaCl, 1% NP-40) supplemented with a protease inhibitor cocktail tablet (Roche Applied Science). The lysis procedure was carried out on ice for 10 minutes with occasional shaking. The lysate was centrifuged at 12000×*g* for 5 minutes and an aliquot of the supernatant (10 µl) was subjected to Western blot analysis either using the anti-VP11 antibody or anti-V5 antibody (Sigma). Sequences of the primers for these plasmids construction used here are listed in Table 2.

**Table 2:** Primer sequences used for the construction of various expression plasmids.

Construct	Primer sequences (5′-3′)
pET-28b/VP11_50−180_	F: GTGGATCCATATAAATTAGACTCTAAATACAC*
	R: TTCAAGCTTAATTGGTAGATTTTTGTGCAAC
pET-28b/VP11-His	F: GCAGGATCCGATGGCACTTTCAAACAATG
	R: GGCCTCGAGTGACAGTGCAACCATTATATTATTATC
pGBKT7-VP11_168−433_	F: TACATATGACGATGGATGAAGTTGTTGCAC
	R: GCCGGATCCCTTATGACAGTGCAACCATT
pDHsp/VP11-V5-His	F: GAGAAGCTTATGGCACTTTCAAACAATGGAG
pDHsp/VP11-FLAG-His	R: GAGCCGCGGTGACAGTGCAACCATTATATTAT
pDHsp/VP24-V5-His	F: CCCAAGCTTATGCACATGTGGGGGGTTTAC
	R:TCCCCGCGGTTTTTCCCCAACCTTAAA
pDHsp/VP26-V5-His	F: CCCAAGCTTAGAAAAATGGAATTTGGCAAC
	R: TCCCCGCGGCTTCTTCTTGATTTCGTCCTTG
pDHsp/VP28-V5-His	F: CCCAAGCTTCTCGTCATGGATCTTTCTTTC
	R: TCCCCGCGGCTCGGTCTCAGTGCCAGAGTAG
pDHsp/VP37-V5-His	F: AGCTAAGCTTCACGATGGCGGTAAACTTGG
	R: CGCCGCGGTGTCCAACAATTTAAAAAG
pDHsp/VP38A-V5-His	F: GCCCGCGGTAAACAATGTCTTCTTCGTCTTC
	R: CGCCGCGGTGAACATGTTACAATTATTC
pDHsp/VP38B-V5-His	F: GCTAAGCTTGTCAGAATGTGCACATTAAAAAC
	R: GTACCGCGGTACTCCACGCTGCTTGG
pDHsp/VP51B-V5-His	F: GCGAAGCTTTAAAAAATGATATTTTATACAATG
	R: GTACCGCGGATCGTCTACCAAATTTTC
pDHsp/VP60B-V5-His	F: GTTGGTACCTTGGTCATGTTCCGTCAATTC
	R: GTACCGCGGAAAGTATAATGGCTGCAC
pDHsp/VP75-V5-His	F: GCTAAGCTTTATACAATGGAGTACATGGAAGAAGGAG
	R: GTACCGCGGAGAAGCATTAGCTCTGATA
pDHsp/VP95-V5-His	F: GTACGGTACCATGCTCAGCTTCAACCCTG
	R: CGA CTCGAGGCCATGGCTAGTGACAAAATG
pDHsp/VP160B-V5-His	F: GTTGGTACCATTACAATGGATATCTTCCTAG
	R: GTACCGCGGTTCTTGAAT GGTGACACTG
pDHsp/VP664-7-V5-His	F: GCTAAGCTTATGACCTATAAGAACTTCCCATC
	R: GTACCGCGGTGCAATAGATGAATCTGG

* The restriction enzyme cutting sites are underlined.

### Fractionation of Virion Proteins by Detergent Treatment at Different NaCl Concentrations

Adult crayfish *Procambarus clarkii* were challenged with WSSV, hemolymph was extracted from the infected crayfish, and the virions were purified from the hemolymph as described by Tsai et al. [7]. The purified virus suspension was treated with 1% Triton X-100 in different concentrations (0, 0.1, 0.5 and 1 M) of NaCl solution, and the soluble and insoluble portions were then fractionated by centrifugation as described previously by Tsai et al. [6]. The intact untreated virion suspension served as a control. The proteins in each of the eight resultant fractions and in the intact purified virion control were resolved by Western blot analysis using the anti-VP11 antibody. The bound antibodies were then stripped out of the membrane, and the membrane was reprobed with antibodies against the WSSV envelope protein VP28, against the tegument protein VP26 and against the nucleocapsid protein VP51C.

### Western Blot Analysis

Protein samples were resolved by SDS-PAGE. After separation, the proteins were transferred to PVDF membranes (MSI). The membranes were incubated in blocking buffer (5% skim milk in TBS [50 mM Tris, 500 mM NaCl, pH 7.5]) at 4°C overnight and then incubated with blocking buffer containing primary antibodies for 1 hour at room temperature. Next, the membrane was washed three times with TBS-T (0.5% Tween 20 in TBS), and incubated with a horseradish peroxidase (HRP)-conjugated secondary antibody. After three more washes, the proteins were visualized using a chemiluminescence reagent (Perkin-Elmer, Inc.).

### Localization of VP11 by Immunoelectron Microscopy (IEM)

Following the method of Tsai et al. [6], aliquots (10 µl) of purified virion suspension were adsorbed onto Formvar-supported, carbon-coated nickel grids (200 mesh) for 5 min at room temperature and then the excess solution was removed. The grids were either prefixed for 5 min with 4% paraformaldehyde and 0.1% Triton X-100 simultaneously in 50 mM Tris buffer (a method modified from Tsai et al., 2006 to disrupt the integrity of the virion envelope) or else were left unfixed and were only incubated with incubation buffer (0.1% Aurion BSA-c™, 15 mM NaN_3_, 10 mM phosphate buffer, 150 mM NaCl, pH 7.4). Other grids were loaded with purified nucleocapsid samples prepared by treating purified virions with detergent and NaCl, and then followed by centrifugal fractionation (see Tsai et al. [6]). These nucleocapsid grids were left unfixed. All grids were then blocked with blocking buffer (5% bovine serum albumin, 5% normal goat serum, 0.1% cold water fish skin gelatin [Aurion], 10 mM phosphate buffer, 150 mM NaCl, pH 7.4) for 15 min and then incubated for 1 h at room temperature with anti-VP11 antibody diluted 1∶50 in the incubation buffer. As a control, an additional grid containing the integrity disrupted virions was incubated using a dilution of preimmune rabbit serum instead of the anti-VP11 antibody. After several washes with incubation buffer, the grids were incubated for 1 h at room temperature with a goat anti-rabbit secondary antibody conjugated with 15 nm diameter gold particles (1∶40 dilution in incubation buffer). The grids were then washed extensively with incubation buffer, washed twice more with distilled water to remove excess salt, and stained with 2% phosphotungstic acid (pH 7.4) for 30 s. Specimens were examined with a transmission electron microscope JEOL JEM1010.

### Membrane Topology of VP11

Based on the deduced amino acid sequence of VP11, its hydropathic profile was predicted by a computer-assisted procedure that followed the methods of Kyte and Doolittle [30] (http://www.expasy.org/). Topology predictions were made using the programs TMHMM (http://www.cbs.dtu.dk/services/TMHMM-2.0/) [31] and SOSUI (http://bp.nuap.nagoya-u.ac.jp/sosui/sosuiframe0.html) [32]. All these methods were used in single sequence mode and all user adjustable parameters were left at their default values.

To further investigate the membrane topology of VP11, indirect immunofluorescence assays were performed on recombinant VP11 that was expressed in Sf9 insect cells. For DNA transfection, Sf9 cells were seeded onto cover glasses placed in a 30 mm dish (8×10^5^ cells), grown in Sf-900 II serum-free medium (Invitrogen Corp.) overnight at 27°C, and the *vp11*-containing plasmids, pDHsp/VP11-V5-His, were transfected into the cells. After transfection and heat shock as described above, the monolayers were washed with PBS, and the cells were fixed in paraformaldehyde (4% in PBS) for 10 minutes at 4°C. Some cells were then treated with 0.1% Triton X-100 in 4% paraformaldehyde/PBS solution for 3 minutes at 4°C while other cells were left untreated. All the cells were then washed thoroughly two times with PBS. After blocking in blocking buffer (5% bovine serum albumin and 2% normal goat serum in PBS) for 16 hours at 4°C, the cells were treated with a polyclonal rabbit anti-V5 antibody (Sigma) for 3 hours at room temperature. Next, the cells were washed 3 times (10 minutes each time) with PBST (0.2% Tween 20 in PBS) and reacted with carboxymethylindocyanine (Cy3) dye-conjugated donkey anti-rabbit IgG antibody (Jackson ImmunoResearch) for 2 hours at room temperature. Counterstaining of the nucleus was performed with 4′-6′-diamidino-2-phenylindole dihydrochloride (DAPI) (Vector Laboratories). After washing three times (10 min each time) with PBST, the cover glasses were wet mounted with anti-fade mounting media (Fluka). During all of the above procedures, the cells were kept in darkness. Fluorescence signals were examined using a confocal scanning laser microscope (Leica TCS SP5).

The membrane topology of VP11 in the WSSV virion was investigated by following the method of Zhu and Yuan [33]. Briefly, aliquots (5 µg of total protein) of purified virions were treated with trypsin (5 µg/ml) (Promega) in 100 µl of buffer (50 mM Tris-HCl [pH 7.5], 1 mM CaCl_2_, 100 mM NaCl) at 37°C for 2 hours. Trypsin digestion was terminated by adding phenylmethylsulfonyl fluoride to a final concentration of 0.5 mM and then adding 1/50 volume of protease inhibitor. In some samples, prior to trypsin digestion, Triton X-100 was added to a final of concentration of 1% to dissolve the viral envelope and expose the internal structure to the protease. Samples were analyzed by Western blotting using antibodies against VP11, and also against the envelope protein VP28 and the tegument protein VP26 for purposes of comparison.

### WSSV VP11 Cloning and Expression in Yeast

To identify the potential candidates of WSSV structural proteins that may interact with WSSV VP11, protein-protein interaction assays were performed using the Matchmaker™ Gold yeast two-hybrid system (Clontech) according to the manufacturer’s protocol. The bait plasmid pGBKT7-VP11 was constructed by cloning the PCR-amplified, WSSV VP11 coding region into the SmaI/BamHI sites of the pGBKT7 DNA BD cloning vector (Clontech) in frame with the GAL4 DNA binding domain. Sequences of the primers used for the pGBKT7-VP11 construction are listed in Table 2. The resulting bait plasmid was transformed into yeast (*Saccharomyces cerevisiae*) strain Y2Hgold. Western blotting with an anti-c-Myc antibody (abcam) probe was used to confirm the expression of the GAL4 DNA-BD fused WSSV VP11 bait protein in lysates from the transformed yeast cells.

### Yeast Two-hybrid Assay

A prey library was constructed by respectively cloning 49 WSSV structural protein and 3 *P. monodon* cellular protein genes (Table 3) into the yeast GAL4 activation domain (GAL4-AD) vector pGADT7 (Clontech). The resultant plasmids were then transformed into the *S. cerevisiae* host strain Y187. The prey DNA inserts were checked by colony PCR followed by DNA sequencing. To identify proteins that interacted with VP11, the prey library was screened by mating with the bait (i.e. the pGBKT7-VP11-transformed Y2Hgold) as per the Matchmaker™ Gold yeast two-hybrid system (Clontech) protocol. Positive and negative controls were created by mating the pGADT7-T-transformed Y187 prey with pGBKT7-53- or pGBKT7-Lam-transformed bait, respectively; the corresponding plasmids were provided by the manufacturer (Clontech). Positive clones that expressed prey proteins that interacted with WSSV VP11 (bait) were selected on a minimal synthetically defined (SD) double-dropout (DDO; SD medium without Leu and Trp [SD/-Leu/-Trp]) medium supplemented with 5-bromo-4-chloro-3-indolyl-α-D-galactopyranoside (X-α-Gal) and Aureobasidin A (DDO/X/A). Blue colonies that grew on a DDO/X/A medium were patched onto higher stringency quadruple-dropout (QDO) (SD medium without Ade, His, Leu, and Trp [SD/-Ade/-His/-Leu/-Trp]) plates containing X-α-Gal and Aureobasidin A (QDO/X/A). The bait protein was tested for autoactivation and toxicity before the two-hybrid screening, and it was confirmed that the GAL4 DNA-BD fused WSSV VP11 does not autonomously activate the reporter genes in Y2HGold in the absence of a prey protein; nor is it toxic to the yeast cells.

**Table 3:** Content of the WSSV structural protein genes yeast two-hybrid prey library constructed in the present study.

WSSV (AF440570)ORF No.	Protein name	Localization on virion	WSSV (AF440570)ORF No.	Protein name	Localization on virion
WSSV019	VP35	NC	WSSV324	VP53C	NC
WSSV027	VP362	nd	WSSV326	VP136A	NC
WSSV051	VP448	nd	WSSV339	VP13A	nd
WSSV052	VP180	Env	WSSV349	VP14	nd
WSSV058	VP24	nd	WSSV359	VP22	nd
WSSV065	VP12	Teg	WSSV362	VP39A	Teg
WSSV067	VP53A,	Env	WSSV364	VP51, VP51C	NC
WSSV092	VP110	Env	WSSV367	VP26	Teg
WSSV094	VP160B	nd	WSSV377	VP16, VP13B	Env
WSSV134	VP36A	Teg	WSSV381	VP56, VP60A	Env
WSSV171	VP53B	nd	WSSV383	VP90	Env
WSSV192	VP337	nd	WSSV388	VP75	NC
WSSV253	VP32	Env	WSSV394	VP11	Env
WSSV254	VP320	nd	WSSV395	VP39, VP39B	Env
WSSV264	VP187	Env	WSSV396	VP31	Env
WSSV269	VP15	NC	WSSV419	VP664	NC
WSSV271	VP124	Env	WSSV445	VP12B	Env
WSSV275	VP73, VP76	NC	WSSV449	VP38B	Env
WSSV293	VP41A	Env	WSSV473	VP19	Env
WSSV294	VP51A	Env	WSSV474	VP60B	NC
WSSV298	VP41B	Env	WSSV480	VP28	Env
WSSV304	VP216	nd	WSSV502	VP95	Teg
WSSV309	VP36B, VP37	NC	WSSV524	VP136B	nd
WSSV311	VP51B, VP52B	Env		WSV010*	nd
WSSV314	VP38, VP38A	Env			

* This ORF was reported in the WSSV China isolate (GenBank accession number AF332093), but it was not predicted to be an ORF in the WSSV TW genome (GenBank accession number AF440570).

NC: nucleocapsid, Env: envelope, Teg: tegument; nd: not yet determined or the protein’s status is still controversial.

** Three *P. monodon* cellular protein genes not listed in the above Table include the pmCBP (chitin-binding protein [54]), pmRACK1 (receptor for activated C kinase-1, GenBank accession number EF569136), and pmRab7 (GenBank accession number DQ231062) which had been reported related to WSSV infection are also included in the yeast two-hybrid prey library and were assayed in the present study.

### Co-immunoprecipitation Assay

Full-length WSSV *vp11* and the genes of the VP11 interaction candidates were inserted into V5- or FLAG-tagged vectors containing the heat-inducible *Drosophila* heat shock protein 70 promoter (pDHsp/V5-His and pDHsp/FLAG-His [29]) by PCR cloning, which used WSSV genomic DNA as the template. For DNA transfection, Sf9 insect cells were seeded onto a 6-well plate (8×10^5^ cells/well) and grown overnight. Plasmids containing the appropriate genes (including the empty vector) were transfected into the Sf9 cells and heat shocked and lysed as described above. An aliquot of the supernatant of the lysate (10 µl) was reserved for Western blot analysis to confirm the expression of the transfected genes. The remaining supernatant (90 µl) was then incubated with 15 µl of anti-FLAG M2 affinity gel (Sigma) at 4°C overnight with rotation. The gel was then washed five times in a 150 µl NP-40 lysis buffer. Aliquots of the total cell lysates and immunoprecipitated complexes were separated by 15% SDS-PAGE and transferred to PVDF membrane. V5-tagged fusion proteins were detected with rabbit anti-V5 antibody and goat anti-rabbit IgG-HRP conjugate (Sigma). FLAG-tagged VP11 and VP11 interaction candidate proteins were detected with mouse anti-FLAG monoclonal antibody (Sigma) and goat anti-mouse IgG-HRP conjugate (Sigma). Sequences of the primers used here are listed in Table 2.

### Oligomerization Assay

To detect the homotypic interaction of the VP11 protein, the VP11 coding region was cloned into pET-28b (+) and the resulting plasmid, pET-28b/VP11-His, was then transformed into BL21 and induced for protein expression, and a modified glutaraldehyde cross-linking method developed by Fadouloglou et al. [34] was used. In brief, the clear lysates prepared from *E. coli* that expressed VP11 protein were mixed with Ni-NTA beads at 4°C for 1 h. After washing extensively with washing buffer (50 mM Tris-HCl, pH 8.0, 300 mM NaCl, 50 mM immidazole, 1 mM DTT), the beads were packed into a column and washed twice with PBS by gravity. Glutaraldehyde solution (0.05% in PBS) was passed through the column, and this was followed by washing with 0.5 M Tris-HCl, pH 8.0. The bound proteins were eluted with elution buffer (50 mM Tris-HCl, pH 8.0, 300 mM NaCl, 250 mM immidazole), and subjected to immunoblot analysis with anti-His antibody (Sigma). Sequences of the primers used for the pET-28b/VP11-His construction are listed in Table 2.

## Results

### Temporal Transcription Analysis of *vp11*

**Figure 1:**
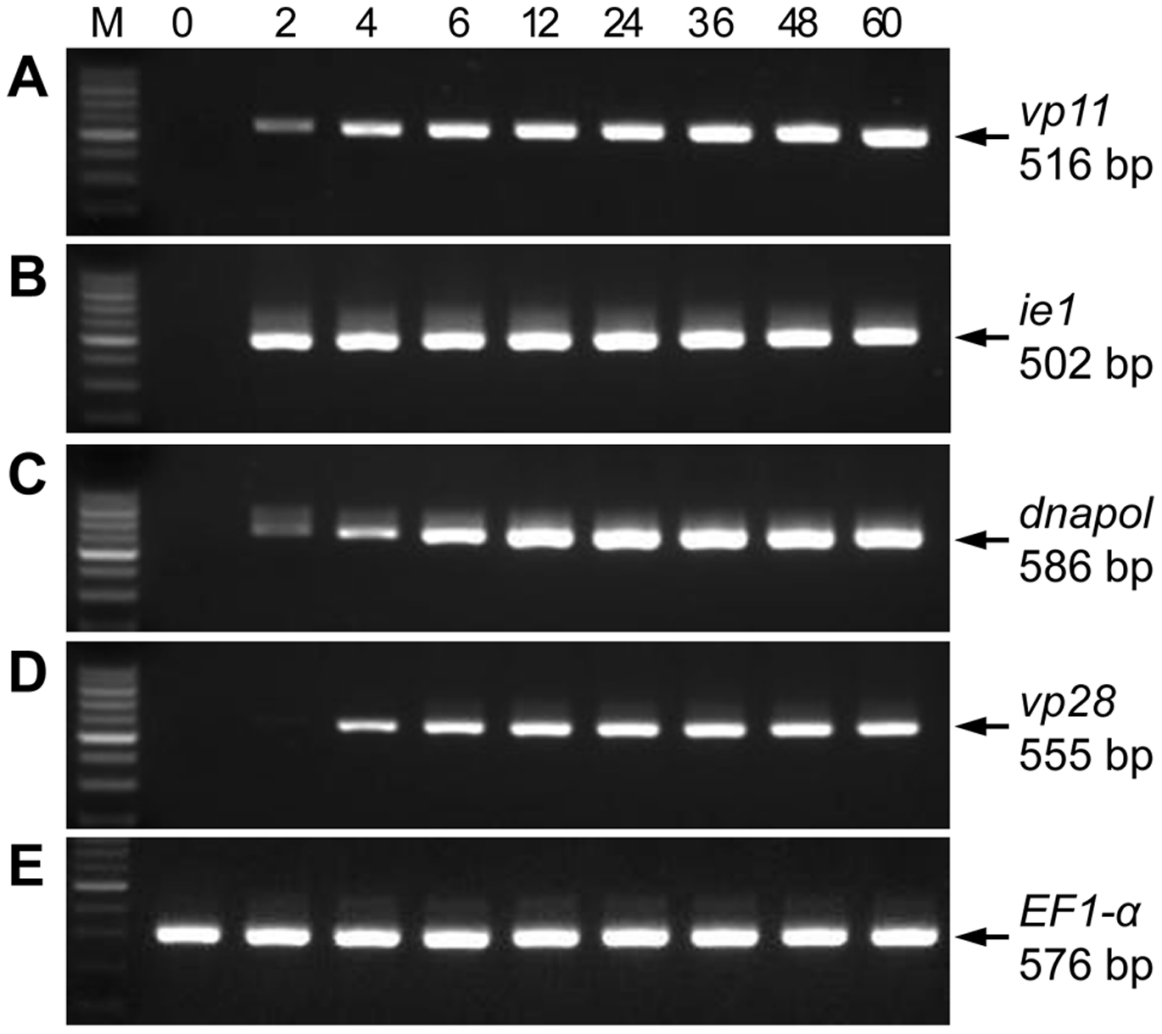
Time course analysis of WSSV *vp11* transcripts by RT-PCR. (A)–(E) Total RNAs were extracted from the gill of WSSV-infected shrimp and subjected to RT-PCR analysis with the indicated primers. Shrimp *elongation factor 1-α* was included as a template control. Lane headings show times post-infection (hours). M: 100 bp DNA ladder. The arrows indicate the size of the amplicon for each gene.

The expression profile of *vp11* in gills of *P. monodon* at various stages of WSSV infection were analyzed by RT-PCR (Fig. 1). The *vp11* transcript was first detected at 2 hpi. Transcription levels approached maximum at 6 hpi and remained high until 60 hpi. By comparison, the immediate early gene *ie1* and the early gene *dnapol* were both transcribed as early as 2 hpi and continued to be found until 60 hpi. The transcript of the WSSV envelope protein gene *vp28* was detected at 4 hpi, and from 6 hpi onward levels were consistently high until the end of the analysis. The *EF1-α* control confirmed the quality of the total RNA templates (Fig. 1E) and another control using a WSSV genomic DNA-specific primer pair, IC-F2/IC-R3 [35], which was derived from an intergenic region of the WSSV genome, confirmed that there was no WSSV DNA contamination (data not shown). From this data we conclude that *vp11* is an early gene.

### Localization of VP11 in the Virion

**Figure 2:**
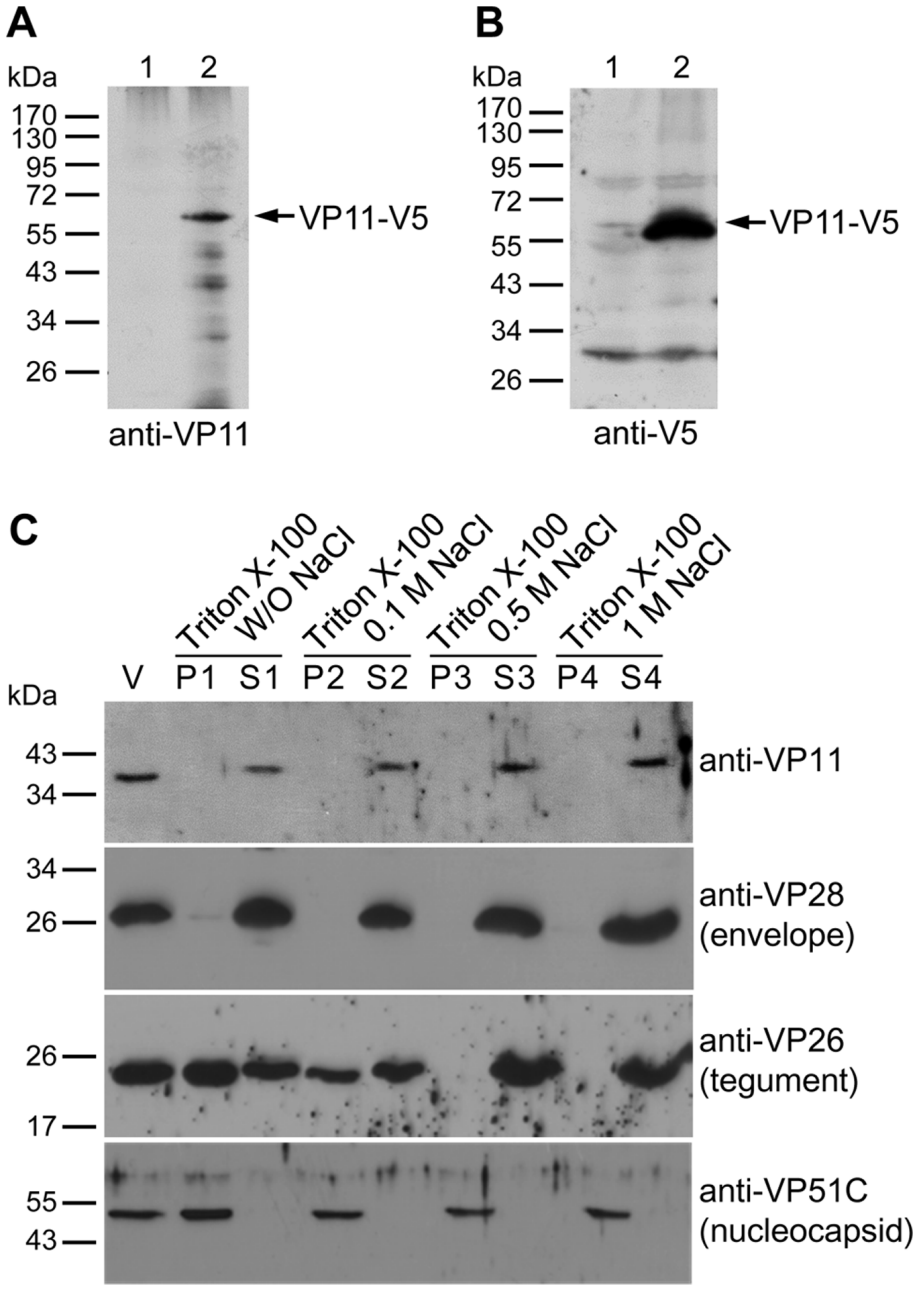
Determination of VP11’s location in the WSSV virion. Western blot analysis of the full-length recombinant WSSV VP11 (VP11-V5) expressed in Sf9 cells using either (A) anti-VP11 antibody or (B) anti-V5 antibody. Lane 1: cell lysate of pDHsp/V5-His transfected Sf9 cells. Lane 2: cell lysate of pDHsp/VP11-V5-His transfected Sf9 cells. (C) Intact WSSV virions were subjected to detergent and NaCl treatment as indicated. After fractionation, the pellet (P) and supernatant (S) fractions were separated on SDS-PAGE and detected by Western blotting to produce profiles that are characteristic of envelope, tegument and nucleocapsid proteins (6). Three representative WSSV structural proteins are shown for comparison. Lane V is the untreated purified virus.

**Figure 3:**
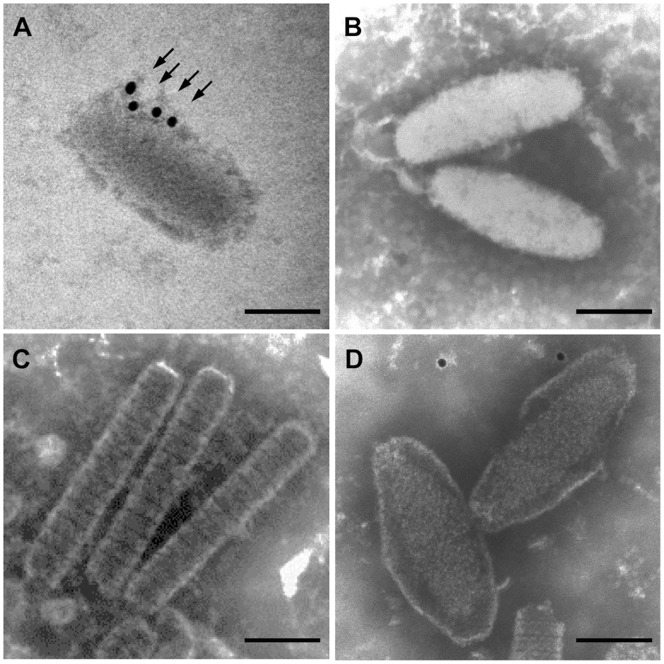
Immunoelectron microscopy of WSSV VP11. Localization of VP11 in the WSSV virion by immunogold assay using a rabbit anti-VP11 antibody probe followed by a gold-labeled secondary antibody. IEMs of (A) the envelope integrity disrupted WSSV virion (B) purified intact virions and (C) the viral nucleocapsids. (D) IEM of the envelope integrity disrupted virions with pre-immune rabbit serum was used as the probe instead of the anti-VP11 antibody. The gold particle signals (arrows) were detected only on the envelope integrity disrupted virion. Scale bars equal 100 nm.

In this study, we used a protein fractionation method developed by Tsai et al. [6] to determine whether VP11 is located in the envelope, the tegument or the nucleocapsid. The specificity of the anti-VP11 antibody was firstly verified by Western blotting performed on SDS-PAGE separated *E. coli* and Sf9 cells expressed recombinant VP11, and WSSV virion proteins (Fig. S1). Figure 2A shows the anti-VP11 antibody successfully recognizing the full-length V5-tagged recombinant WSSV VP11 that was expressed in the Sf9 cells. The same protein band was also recognized by reprobing with the anti-V5 antibody (Fig. 2B). Using Western blot analysis of the eight different fractions of the WSSV virion proteins, VP11’s profile was compared to the profiles of the known envelope, tegument and nucleocapsid proteins (VP28, VP26, and VP51C, respectively; see Fig. 2C). The results show that in the 1% Triton X-100-treated preparations VP11 was similar to the envelope protein VP28 in that it was almost completely soluble in both the presence and absence of NaCl. We, therefore, conclude that VP11 is a WSSV envelope protein. IEM provides more evidence in supporting that VP11 is an envelope protein. When a gold-labeled secondary antibody was used in conjunction with an anti-VP11 antiserum, gold particles were observed on the virion. Although, positive signals were only found on the envelope of envelope integrity disrupted virion (Fig. 3A), but not on the intact virion (Fig. 3B) or on the nucleocapsid surface (Fig. 3C), this situation it also demonstrated VP11 is located on the envelope. Control experiments showed that no gold particles were found on the envelope disrupted WSSV virion when normal rabbit serum was used as the primary antibody (Fig. 3D). Collectively, these results confirmed that VP11 is a WSSV envelope protein.

### Membrane Topology of VP11

**Figure 4:**
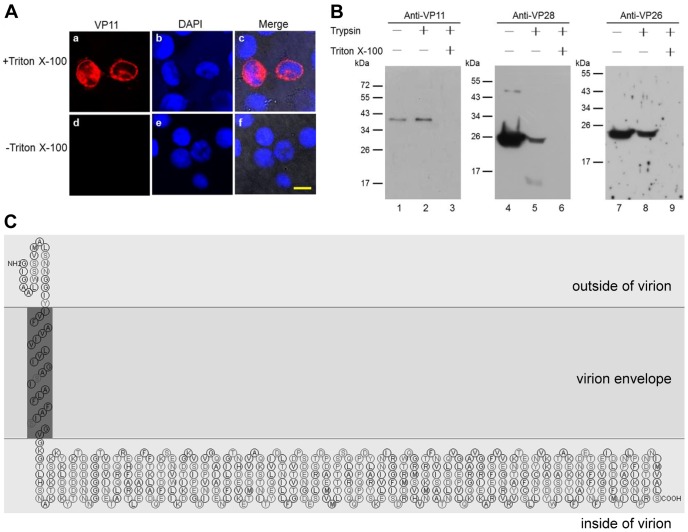
Membrane topology of WSSV VP11. (A) V5-tagged recombinant VP11 (VP11-V5) was transiently expressed in Sf9 cells that were fixed with paraformaldehyde and either permeabilized with Triton X-100 (panels a–c) or not permeabilized (panels d–f). Due to the failure to detect the C-terminal V5 tag in the non-permeabilized cells, we conclude that VP11’s C-terminal must be located inside the cell membrane (cf the PmSTAT assay used by Liu et al. [53]). VP11-V5 was visualized with rabbit anti-V5 antibody and Cy3-conjugated donkey anti-rabbit IgG antibody (panels a and d). Nuclei were visualized by counterstaining with DAPI (panels b and e). Panels c and f show the merged Cy3 and DAPI signals. Scale bar equals 10 µm. (B) Western blot analysis showing trypsin digestion of VP11 and two other proteins for comparison: VP28 (an envelope protein with most of its C-terminal exposed outside the envelope) and VP26 (a tegument protein) in intact and detergent-treated virions. (C) Schematic of the proposed transmembrane topology of VP11. (This schematic was modified from the SOSUI program prediction by transposing the inside and outside of the virion.).

The hydrophobicity profile generated using Kyte-Doolittle hydropathy plot analysis showed a high hydrophobicity in the N-terminal region of VP11 (data not shown). We used two programs, TMHMM and SOSUI, to predict membrane topology, and both programs provided very similar results with only slightly different start and end points for the predicted transmembrane segments: TMHMM and SOSUI predicted that VP11 encodes a transmembrane helix at aa 7–29 and aa 21–43, respectively. To confirm these predictions, a recombinant VP11 fusion protein (VP11-V5) with a V5 tag on its C-terminus was subjected to indirect immunofluorescence assays in transfected Sf9 cells. In cells that were treated with Triton X-100 to render them permeable to the anti-V5 antibody, the full length VP11-V5 was detected both in the plasma membrane region and in the cytoplasm (Fig. 4A, upper panels). However, in the nonpermeabilized cells, the VP11-V5 could no longer be detected either in the cytoplasm or on the outside surface of the plasma membrane (Fig. 4A, lower panels). We therefore conclude that the C-terminal region of the protein must be located inside the cell membrane.

The topology of VP11 in the WSSV virion was investigated by using trypsin to distinguish between proteins that were accessible to proteolysis and those that were protected from digestion by the lipid bilayer. WSSV virions were either left untreated or treated with trypsin in the absence or presence of the detergent Triton X-100, and the digested products were analyzed by Western immunoblotting using antibodies against VP11, VP26 or VP28. As expected, the VP11 antibody recognized the VP11 38 kDa proteins in the untreated virions (Fig. 4B, lane 1), whereas after digestion with trypsin in the presence of Triton X-100, the 38 kDa protein was no longer detected (Fig. 4B, lane 3). However, it is surprising that in the absence of Triton X-100, the trypsin failed to digest the VP11 (Fig. 4B, lane 2). These results suggest that even though Figures 2 and 3 show that VP11 is an envelope protein, in this assay, nevertheless, it was somehow protected from trypsin digestion by the envelope. So far, none of the WSSV envelope protein with large portion(s) toward the inside of virion was reported. For comparison, the treated virions were also subjected to Western blotting to detect the tegument protein VP26 (which is protected from trypsin digestion by the envelope) and the envelope protein VP28, an envelope protein with a portion of its C-terminal expose outside of the virion. As expected, VP28 was digested into two bands in the absence of Triton X-100, and completely digested in the presence of Triton X-100 (Fig. 4B, lanes 4–6); VP26 was only digested in the presence of Triton X-100 (Fig. 4B, lanes 7–9). By comparing all the information and results for membrane topology of VP11, we interpret this data to mean that VP11 is a transmembrane protein, which contrary to the predictions of the TMHMM and SOSUI programs, has its C-terminal inside the virion envelope. A schematic of the proposed transmembrane topology of VP11 is shown in Figure 4C. This Figure shows the result predicted by the SOSUI program, but the inside and outside is transposed.

### Screening for VP11 Interaction Proteins

**Figure 5:**
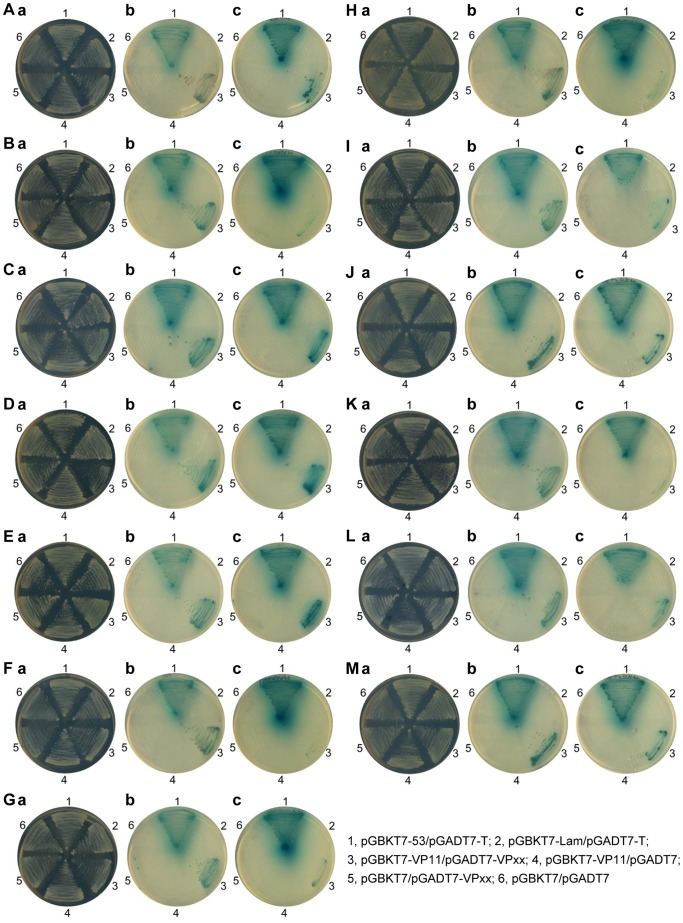
Yeast two-hybrid screening results for all of the confirmed WSSV VP11 interaction proteins. (A) to (M) VP11’s interactions with VP24, VP26, VP28, VP37, VP38A, VP38B, VP51B, VP60B, VP75, VP95, VP160B, VP664-7 and itself, respectively. (a) Yeast growth on medium lacking both Leu and Trp (DDO) indicates the presence of each respective pair of plasmids. (b) and (c) Yeast growth on low stringency (DDO/X/A) and high stringency (QDO/X/A) medium, respectively. The blue signals in (b) and (c) are due to the presence of X-α-Gal. The positive signals represent protein-protein interactions. The numbers around the plates indicate the bait and prey plasmids of the transformed yeast: 1, pGBKT7-53/pGADT7-T; 2, pGBKT7-Lam/pGADT7-T; 3, pGBKT7-VP11/pGADT7-VPxx; 4, pGBKT7-VP11/pGADT7; 5, pGBKT7/pGADT7-VPxx; 6, pGBKT7/pGADT7, with VPxx respectively standing for each of the WSSV structural proteins listed above.

An auto-activation bait protein test found that GAL4 DNA-BD-fused full-length WSSV VP11 autonomously activated the reporter genes in Y2HGold in the absence of a prey protein, and that this autonomous activation activity was due to its N-terminus segment (data not shown). We, therefore, constructed a VP11 subclone with a truncated N-terminal (aa 168–433) (Table 2), which was then used as bait to screen the prey library. Yeast two-hybrid mating revealed a total of 18 WSSV structural proteins that interacted with VP11: in addition to itself, these included VP12, VP24, VP26, VP28, VP37, VP38A, VP38B, VP39A, VP51B, VP51C, VP60B, VP75, VP136A, VP136B, VP160B, VP192, and VP664. Figure 5 shows the 13 WSSV proteins whose interactions with VP11 were successfully confirmed by the co-immunoprecipitation assay are described in the next section.

### Confirmation of the VP11 Interaction Proteins

**Figure 6:**
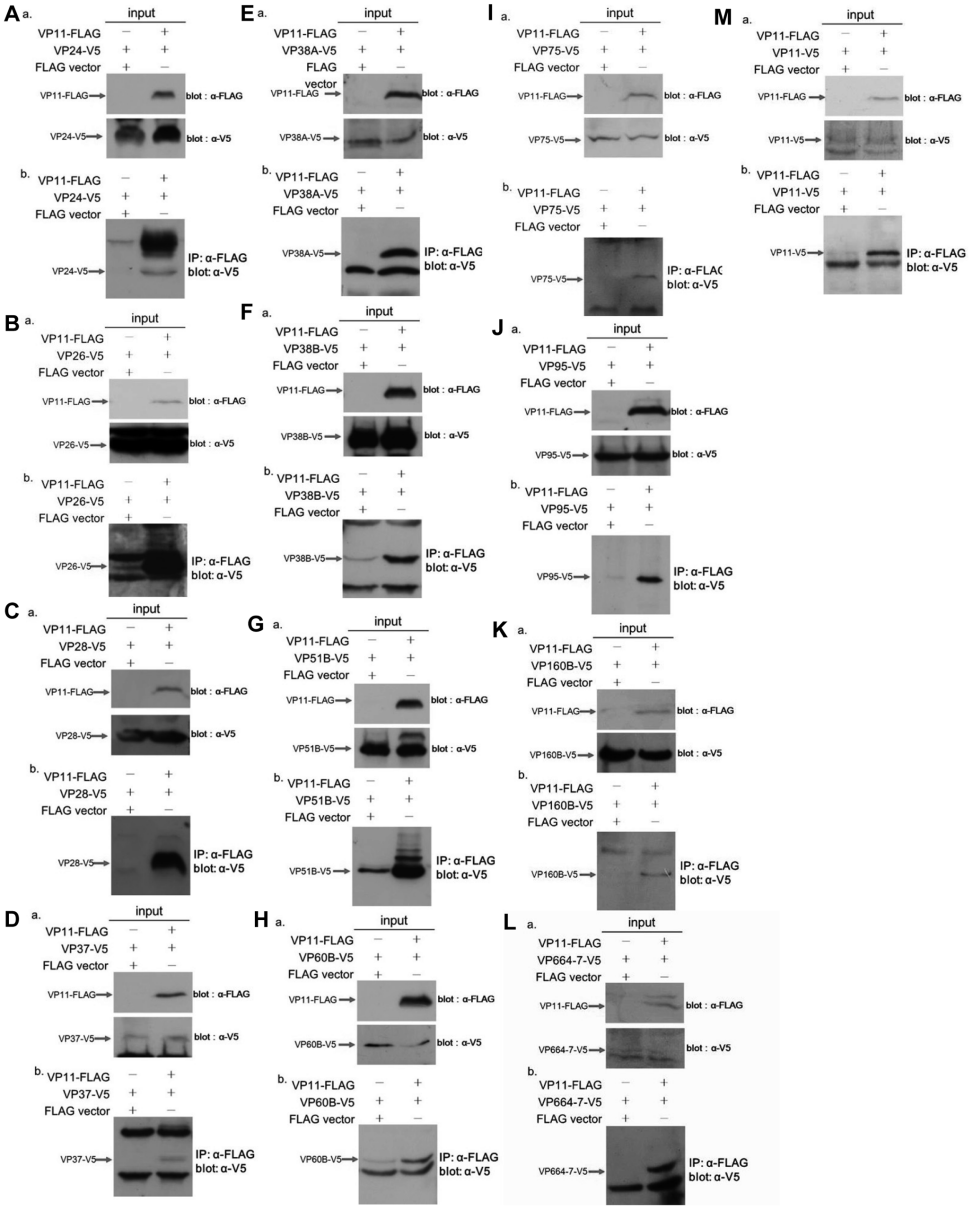
Co-immunoprecipitation confirmation of WSSV VP11 interaction proteins. (A) to (M) co-immunoprecipitation of FLAG-tagged VP11 (VP11-FLAG) with V5-tagged interaction proteins VP24, VP26, VP28, VP37, VP38A, VP38B, VP51B, VP60B, VP75, VP95, VP160B, VP664-7, and VP11. Sf9 cells were co-transfected with FLAG-tagged VP11 plasmids, V5-tagged WSSV structural protein plasmids and empty plasmids (vector) as indicated. At 6 h after heat shock, the cell lysates were harvested. (a) After separation by SDS-PAGE, input expression was confirmed by Western blotting using either anti-V5 antibody or anti-FLAG antibody as a probe. Arrows indicate the expressed VP11-FLAG and respective V5-tagged WSSV structural protein. (b) The cell lysates were immunoprecipitated with anti-FLAG M2 affinity resins and then the immunoprecipitated complexes were subjected to Western blot analysis with an anti-V5 antibody probe.

The interactions between VP11 and its partners revealed by the yeast two-hybrid screening were further confirmed using a co-immunoprecipitation assay in which FLAG-tagged VP11 (VP11-FLAG) and V5-tagged interaction candidates (VPxx-V5) were co-expressed in Sf9 insect cells. As shown in the “a” panels of Figure 6, all of the recombinant tagged proteins were successfully expressed in the Sf9 cells. A pilot experiment confirmed that the VP11-FLAG proteins could be efficiently precipitated by the anti-FLAG antibody, and the binding specificity of VP11-FLAG with anti-FLAG M2 affinity gel was reconfirmed by reacting VP11-FLAG protein with anti-HA antibody-conjugated beads (data not shown). In the co-immunoprecipitation assays, VP24-V5, VP26-V5, VP28-V5, VP37-V5, VP38A-V5, VP38B-V5, VP51B-V5, VP60B-V5, VP75-V5, VP95-V5, VP160B-V5, VP664-7-V5 (a partial fragment of VP664), and VP11 itself (VP11-V5) were co-immunoprecipitated by anti-FLAG M2 affinity gel and detected by Western blotting using anti-V5 antibody (Fig. 6, “b” panels). From these results, we conclude that VP11 formed a complex only with these specific proteins, and that these interactions were independent of the tags.

**Figure 7:**
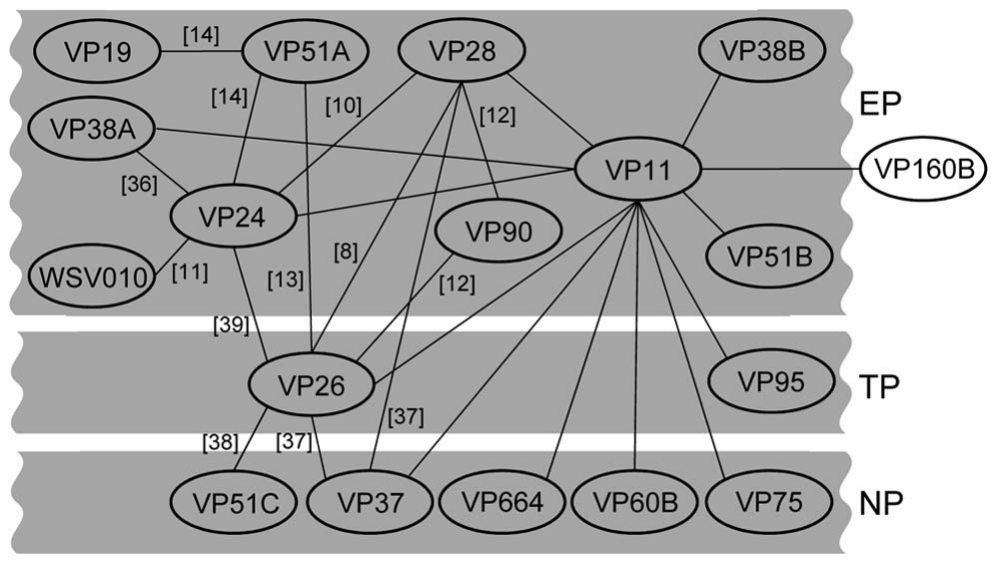
An interactome diagram showing the relationships among the indicated WSSV structural proteins. Although these interactions are not yet fully understood, VP11, VP24, VP26, and VP28 seem to act as the core of the virion protein complex. VP11, VP26, and VP28 interact directly with the nucleocapsid proteins VP664, VP60B, VP75, VP51C, and VP37, respectively, and may serve to anchor the entire envelope protein complex to the nucleocapsid. The interactions shown here were established either in the present study or else were based on previous results as indicated. The location of the unshaded protein (i.e. VP160B) on the virion has not yet been determined. EP: envelope proteins. TP: tegument proteins. NP: nucleocapsid proteins.

Figure 7 is an interactome diagram that uses the protein-protein interactions listed above as well as those reported previously [8], [10]–[14], [36]–[39] to schematically show the known interactions among the WSSV structural proteins.

### VP11 has an Affinity for Self-interaction and Forms Dimers

**Figure 8:**
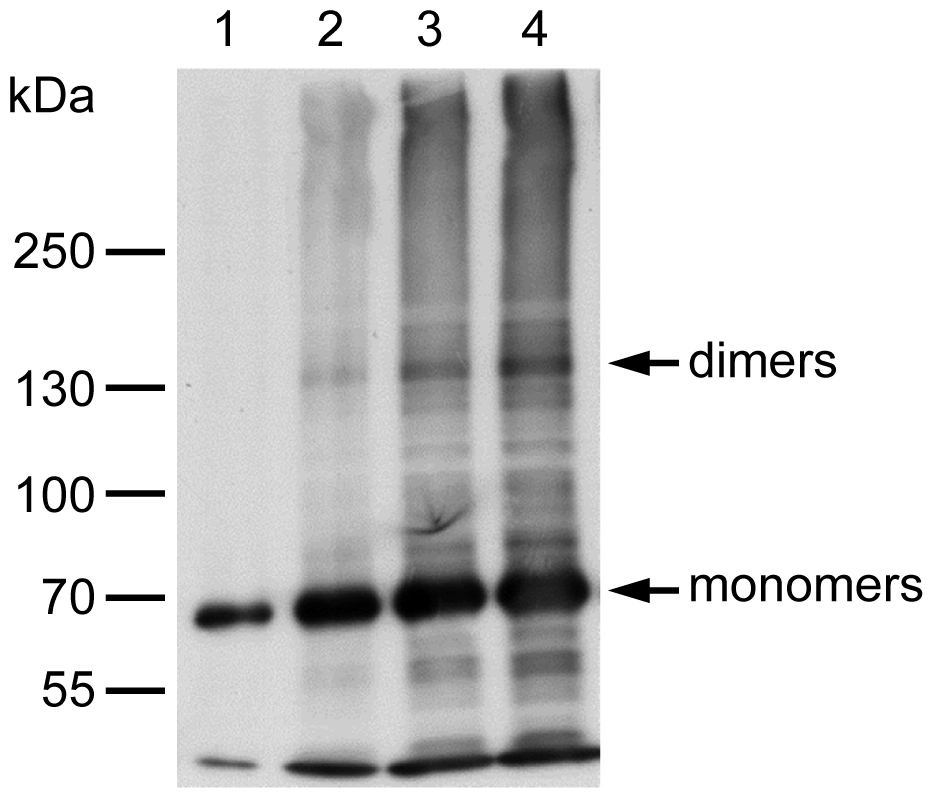
Homotypic interaction of VP11. Immunoblot analysis of glutaraldehyde-treated VP11 proteins immobilized on the Ni-NTA beads. Lane 1: VP11 bound on Ni-NTA beads. Lanes 2–4: First, second, and third elutions from the column of VP11 protein after glutaraldehyde treatment. Monomers and dimers of VP11 proteins are indicated.

Both the yeast two-hybrid and co-immunoprecipitation assays showed that VP11 self-interacted (Figs. 5M and 6M). An approach that implemented a modified glutaraldehyde cross-linking method was used to further analyze VP11’s homotypic interaction. In this method, glutaraldehyde treatment stabilized the oligomerization state of VP11 immobilized to the beads, and then the treated VP11 was eluted, and analyzed by immunoblotting with the anti-His antibody. As shown in Fig. 8, some of the treated VP11 proteins ran as oligomers (dimers) on SDS-PAGE. All these three assays showed that VP11 proteins interact with each other in vitro as well as in yeast and Sf9 cells.

## Discussion

WSSV VP11 was first identified by Tsai et al [7] using mass spectrometry of an 11 kDa band of WSSV virion proteins that had been separated on SDS-PAGE. However, in the WSSV-TW strain, the coding region of *vp11* corresponds to *wssv394*, which translates to a predicted polypeptide of 433 aa with a theoretical molecular mass of 48 kDa. In the present study, a Western blotting analysis of WSSV virion proteins separated by SDS-PAGE gave a size of around 38 kDa for VP11 (Figs. 2C and 4B lanes 1 & 2). The same result was also observed by Kang et al [40] who speculated that *wssv394* encodes an unstable 48 kDa protein, which, after synthesis, is then processed or degraded to the 38 and 11 kDa proteins. However, it seems that this cannot after all be the correct explanation. The short peptide sequence that Tsai et al. [7] identified by tandem mass spectrometry matched the VP11 region from aa 253–260 (J.-M. Tsai, personal communication), which implies that the 11 kDa protein must have been produced by truncation of both the N-terminal and C-terminal in the full-length protein. (Truncation of only the N-terminal would leave a protein with a predicted mass of ∼20 kDa, whereas truncation of only the C-terminal would leave a protein with a predicted size of ∼28 kDa.) Furthermore, since we failed to detect the 11 kDa protein here with an anti-VP11 antibody derived from VP11 aa 50–180, this implies that at least the first 180 aa of full-length VP11’s N-terminal must have been truncated. By considering the data of previous research as well as our own, we, therefore, hypothesize that the 38 kDa and 11 kDa proteins are different, independently degraded forms of WSSV VP11. This kind of truncation has been previously reported for other WSSV structural proteins. For example, the 72 kDa WSSV VP51A protein has been detected in several truncated forms (51 kDa as well as several smaller VP51A proteins) in the WSSV virion proteins [13], whereas Xie et al. [8] identified five different truncated species of VP150 in purified WSSV virions. All of these truncated forms appear to be essential components of the viral particle, and they may all participate in various stages of virus morphogenesis. It therefore seems probable that the 11 kDa truncated form of VP11 might likewise play an important, meaningful, structural role. On the other hand, there are two WSSV structural proteins, the VP38A (also named as VP38, WSSV314) and VP38B (WSSV449), that were identified with a molecular mass around 38 kDa. The possibility of the 38 kDa protein being identified was due to antibody cross-reaction with these two proteins, which also were excluded by sequences alignment (Fig. S2) and Western blot analysis (Fig. S3).

Fractionation analysis of the virion proteins by detergent treatment at different NaCl concentrations identified VP11 as an envelope protein (Fig. 2C). Viral envelope proteins are important because they often play vital roles in cell targeting, virus entry, and assembly and budding [41]–[44]. For instance, in WSSV, the envelope protein VP28 is known to be involved in cell attachment during infection [18], [45]. Determination of a protein’s membrane topology is an important first step toward understanding its function. The protein domains exposed on the surface of viruses can play fundamental roles in infection because they bind to cell receptors promote cell fusion processes, or interact with elements of the host immune system [41], [46], [47]. In the case of VP11, the topological prediction programs TMHMM and SOSUI predicted a transmembrane helix near its N-terminal and a C-terminal region that was outside of the plasma membrane. However, experimental membrane topology assays suggested that the C-terminal region was in fact inside the virion: first, an immunofluorescence assay performed on rVP11 fusion protein expressed in Sf9 showed that the C-terminal V5 epitopes of the rVP11 could only be detected on the inner surface of the cell membranes (Fig. 4A); and furthermore, in the WSSV virions, trypsin digestion of VP11 in the absence of detergent (Fig. 4B, lane 2) also confirmed that the C-terminal of VP11 was on the inside of the virion. In this trypsin digestion assay, a WSSV envelope protein with its C-terminal pointed towards the inside of the virion was not available. The VP28, an envelope protein with a portion of its C-terminal exposed outside of the virion, and the VP26, a tegument protein, were used as controls. The results showed that the trypsin digestion patterns of VP11 was not like VP28 but actually similar to VP26, which was in the absence of detergent and was protected from trypsin digestion by the virion envelope. This topology was also suggested by the IEM assay in which the anti-VP11 antibody derived from recombinant VP11 with a truncated N-terminus failed to detect VP11 on the surface of intact virions (Fig. 3B) but VP11 was detected on envelope integrity disrupted virions (Fig. 3A). We therefore conclude that a large portion of the VP11 protein must be embedded in the virion (Fig. 4C). Meanwhile, the protein-protein interaction screening analysis further showed that VP11 interacted with several WSSV proteins (VP26, VP28, and VP37) that do directly interact with the host cell [22], [48], [49]. This in turn implies that VP11 might also participate indirectly in virus targeting and entrance. Although the structural relationship between WSSV’s three layers, the envelope, tegument and nucleocapsid, has not yet been clearly established, we found here that VP11 interacts with VP664, which is the major nucleocapsid protein of WSSV. This is the first WSSV structural protein that has been shown to interact with VP664. VP11 was also found to interact with other nucleocapsid proteins, VP37, VP60B, and VP75. In addition, since VP11 also interacted with the tegument protein VP26, it must also indirectly interact with another nucleocapsid protein, VP51C. Given that VP11 also interacted with several envelope proteins (see Fig. 7), we hypothesize that VP11 might act to anchor the envelope to the nucleocapsid.

Self-interaction has already been reported in several other WSSV structural proteins, such as VP19, VP24, VP26, VP28, and VP51A [14], [39], [50], and here we show that VP11 is able to form dimers (Fig. 8). In addition, as noted in the yeast two-hybrid screening results, VP11 is capable of autoactivation (data not shown). A pilot experiment also demonstrated that the N-terminal region of VP11 had transactivation activity (data not shown). Both of these characteristics suggest that VP11 might be a transcription factor [51]. In addition, some other virus structural proteins have also been reported to act as transcription factors. For example, herpes simplex virus (HSV) VP16 plays a key role in regulating the transcription of HSV immediate-early genes at the beginning of the viral infection cycle [52]. Furthermore, the temporal transcription analysis in Figure 1 shows that *vp11* is an early gene, with a transcript that was first detected at 2 hpi, and with transcription levels approaching a maximum at 6 hpi and remaining high until 60 hpi. All of these findings suggest that VP11 might act as a transcriptional factor that plays a significant role in the early stage of virus infection.

In summary, we have shown that VP11 is an important molecule that is possibly involved in many stages of the WSSV infection cycle, not only in virion assembly, but also in regulating viral gene expression. The WSSV virion is a complex structure, and this study of VP11’s interactome has revealed several meaningful aspects of WSSV’s structural composition. The data produced by this research also suggests that VP11 might be a potential target for developing anti-WSSV strategies.

## Supporting Information

Figure S1 **Anti-VP11 antibody verification and molecular mass comparison of VP11 proteins.** Western blot analysis of *E. coli* and Sf9 cells expressed WSSV VP11 proteins with anti-VP11 antibody. Lanes 1 and 2: lysates of pET-28b (+), and pET-28b/VP11-His transformed *E. coli*; lanes 3 and 4: lysates of pDHsp/V5-His, and pDHsp/VP11-V5-His transfected Sf9 cells, respectively. Lane 5: WSSV virion proteins. The recombinant and virion VP11 proteins (rVP11 and WSSV VP11, respectively) are indicated by arrows.
(TIF)

Figure S2 **Amino acid sequences similarity of WSSV VP11, VP38A, and VP38B.** Multiple sequence alignment of deduced amino acid sequence of WSSV VP11, and the sequences of two other WSSV structural proteins, the VP38A (also named as VP38, WSSV314) and VP38B (WSSV449), respectively that were identified with molecular a mass around 38 kDa.
(TIF)

Figure S3 **Analysis on the cross-reaction of anti-VP11 antibody with WSSV VP38A and VP38B.** Western blot analysis of the cross-reaction of anti-VP11 antiby with WSSV VP38A and VP38B. Lysates of pDHsp/V5-His, pDHsp/VP11-V5-His, pDHsp/VP38A-V5-His, pDHsp/VP38B-V5-His transfected Sf9 cells (lanes 1 to 4, respectively), and WSSV virion proteins (lanes 5) were separated on SDS-PAGE and blotted with anti-VP11 antibody (A). The same loadings were also analyzed with anti-V5 antibody to confirm the successful expression of each recombinant protein (B).
(TIF)
