# Supplementary figures and images for: Characterization and Interactome Study of White Spot Syndrome Virus Envelope Protein VP11
Source: PLoS One. 2014 Jan 21;9(1):e85779. doi: 10.1371/journal.pone.0085779 (PMC3897518; doi:10.1371/journal.pone.0085779)

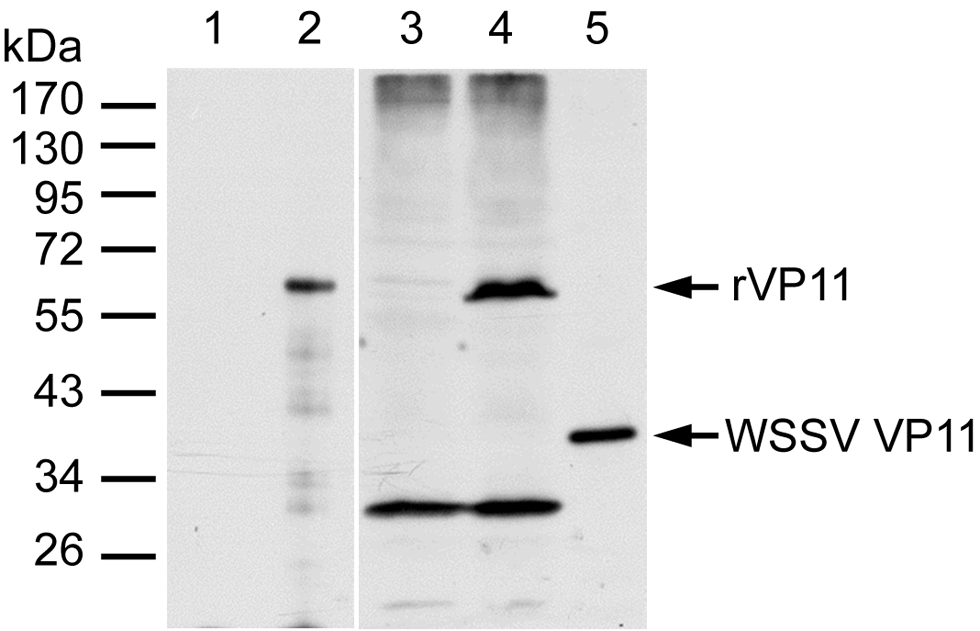

Supplement: Figure S1 — Anti-VP11 antibody verification and molecular mass comparison of VP11 proteins. Western blot analysis of E. coli and Sf9 cells expressed WSSV VP11 proteins with anti-VP11 antibody. Lanes 1 and 2: lysates of pET-28b (+), and pET-28b/VP11-His transformed E. coli; lanes 3 and 4: lysates of pDHsp/V5-His, and pDHsp/VP11-V5-His transfected Sf9 cells, respectively. Lane 5: WSSV virion proteins. The recombinant and virion VP11 proteins (rVP11 and WSSV VP11, respectively) are indicated by arrows. (TIF) [file pone.0085779.s001.tif]

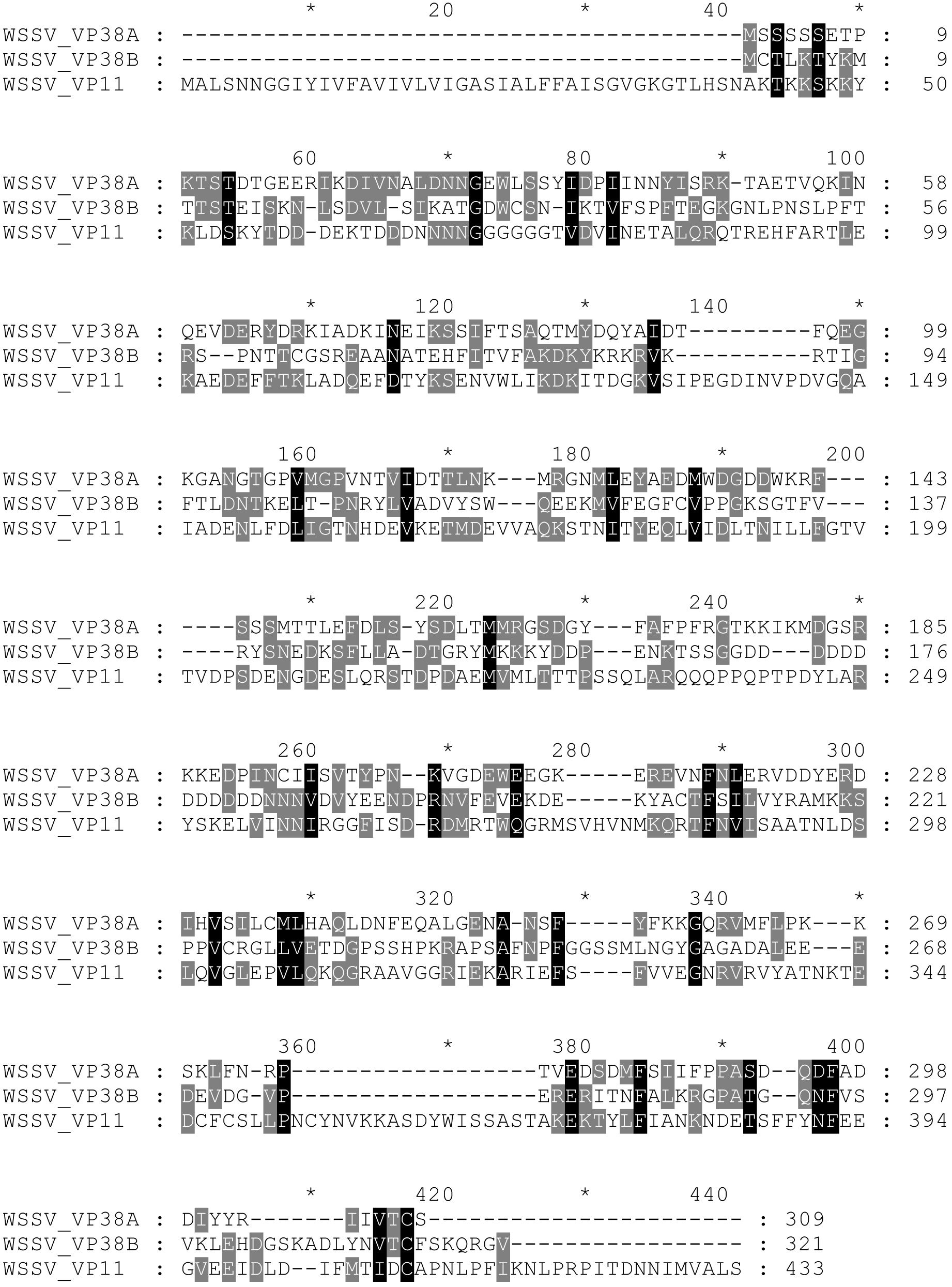

Supplement: Figure S2 — Amino acid sequences similarity of WSSV VP11, VP38A, and VP38B. Multiple sequence alignment of deduced amino acid sequence of WSSV VP11, and the sequences of two other WSSV structural proteins, the VP38A (also named as VP38, WSSV314) and VP38B (WSSV449), respectively that were identified with molecular a mass around 38 kDa. (TIF) [file pone.0085779.s002.tif]

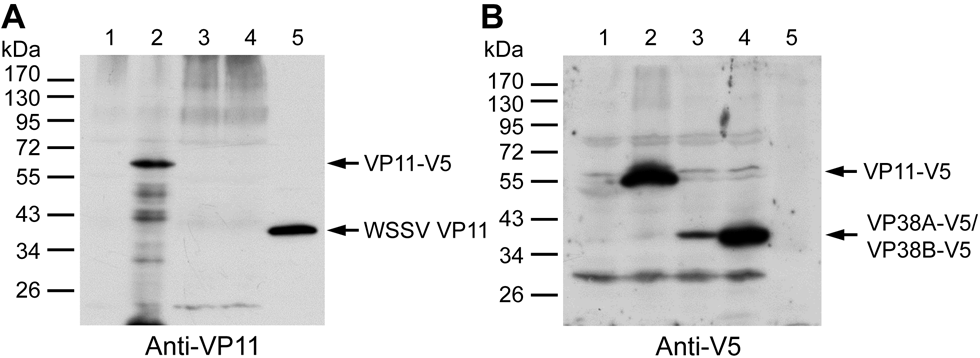

Supplement: Figure S3 — Analysis on the cross-reaction of anti-VP11 antibody with WSSV VP38A and VP38B. Western blot analysis of the cross-reaction of anti-VP11 antiby with WSSV VP38A and VP38B. Lysates of pDHsp/V5-His, pDHsp/VP11-V5-His, pDHsp/VP38A-V5-His, pDHsp/VP38B-V5-His transfected Sf9 cells (lanes 1 to 4, respectively), and WSSV virion proteins (lanes 5) were separated on SDS-PAGE and blotted with anti-VP11 antibody (A). The same loadings were also analyzed with anti-V5 antibody to confirm the successful expression of each recombinant protein (B). (TIF) [file pone.0085779.s003.tif]
